# Low apolipoprotein A-I levels in Friedreich’s ataxia and in frataxin-deficient cells: Implications for therapy

**DOI:** 10.1371/journal.pone.0192779

**Published:** 2018-02-15

**Authors:** QingQing Wang, Lili Guo, Cassandra J. Strawser, Lauren A. Hauser, Wei-Ting Hwang, Nathaniel W. Snyder, David R. Lynch, Clementina Mesaros, Ian A. Blair

**Affiliations:** 1 Penn/CHOP Center of Excellence in Friedreich’s Ataxia, The Children’s Hospital of Philadelphia, Philadelphia, Pennsylvania, United States of America; 2 Penn SRP Center and Center of Excellence in Environmental Toxicology Center, Department of Systems Pharmacology and Translational Therapeutics, Perelman School of Medicine, University of Pennsylvania Philadelphia, Philadelphia, Pennsylvania, United States of America; 3 Division of Neurology, The Children’s Hospital of Philadelphia, Philadelphia, Pennsylvania, United States of America; 4 Department of Biostatistics, Epidemiology, and Informatics, Perelman School of Medicine, University of Pennsylvania, Philadelphia, Pennsylvania, United States of America; 5 AJ Drexel Autism Institute, Drexel University, Philadelphia, Pennsylvania, United States of America; Louisiana State University Health Sciences Center, UNITED STATES

## Abstract

Friedreich’s ataxia (FA) is an autosomal recessive neurodegenerative disorder, which results primarily from reduced expression of the mitochondrial protein frataxin. FA has an estimated prevalence of one in 50,000 in the population, making it the most common hereditary ataxia. Paradoxically, mortality arises most frequently from cardiomyopathy and cardiac failure rather than from neurological effects. Decreased high-density lipoprotein (HDL) and apolipoprotein A-I (ApoA-l) levels in the general population are associated with an increased risk of mortality from cardiomyopathy and heart failure. However, the pathophysiology of heart disease in FA is non-vascular and there are conflicting data on HDL-cholesterol in FA. Two studies have shown a decrease in HDL-cholesterol compared with controls and two have shown there was no difference between FA and controls. One also showed that there was no difference in serum Apo-A-I levels in FA when compared with controls. Using a highly specific stable isotope dilution mass spectrometry-based assay, we demonstrated a 21.6% decrease in serum ApoA-I in FA patients (134.8 mg/dL, n = 95) compared with non-affected controls (172.1 mg/dL, n = 95). This is similar to the difference in serum ApoA-I levels between non-smokers and tobacco smokers. Knockdown of frataxin by > 70% in human hepatoma HepG2 cells caused a 20% reduction in secreted ApoA-I. Simvastatin, a 3-hydroxy-3-methylglutaryl-coenzyme A (HMG-CoA) reductase inhibitor caused a 200% increase in HMG-CoA in the control HepG2 cells with a similar increase in the frataxin knockdown HepG2 cells, back to levels found in the control cells. There was a concomitant 20% increase in secreted ApoA-I to levels found in the control cells that were treated with simvastatin. This study provides compelling evidence that ApoA-I levels are reduced in FA patients compared with controls and suggest that statin treatment would normalize the ApoA-I levels.

## Introduction

Friedreich’s ataxia (FA) is an autosomal recessive neurodegenerative disorder, which results primarily from reduced expression of the mitochondrial protein frataxin [1–3]. Although the exact mechanism of frataxin action has not been completely defined, the protein is involved in the assembly of iron-sulfur clusters, which are important for optimal mitochondrial function [4]. Progressive ataxia is a characteristic feature of FA [5], although a significant number of patients also experience hypertrophic cardiomyopathy that eventually progresses to heart failure. In fact, heart failure due to cardiomyopathy rather than neurodegeneration, is the primary cause of death in FA [6–9]. The heart typically maintains adequate systolic function [10] in FA patients who develop a severe hypertrophic cardiomyopathy until shortly before death [11]. The hypertrophy is associated with a striking proliferation of mitochondria within the cardiomyocytes, coupled with significant loss of contractile fibers [12,13]. Impaired myocardial perfusion reserve, fibrosis and iron overload represent early manifestations of cardiomyopathy in FA prior to left ventricular hypertrophy [6]. It was suggested that these effects could be due to subclinical myocardial disease in FA caused by mitochondrial cardiomyopathy with metabolic syndrome [6].

**Fig 1:**
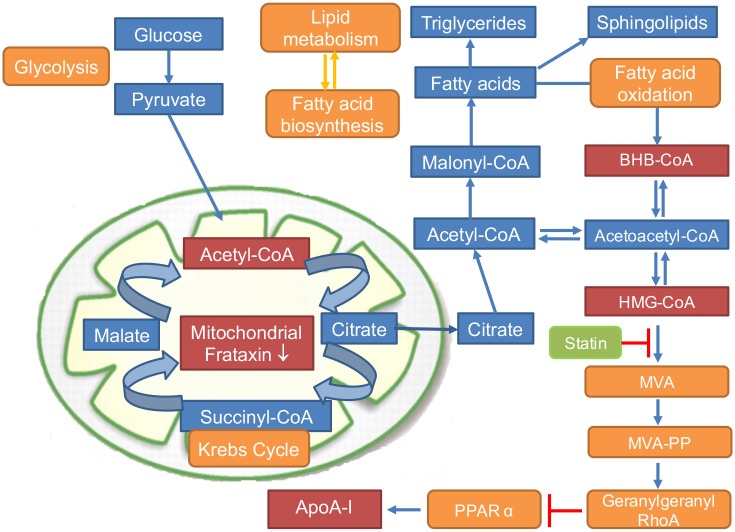
Schematic showing changes in glycolysis, lipid metabolism, and fatty acid biosynthesis/oxidation, and ApoA-I expression in FA.

Under normal circumstances, fatty acids are the predominant energetic substrates for the heart, with β-oxidation providing 50% to 70% of myocardial ATP need [14]. After transport into cardiomyocytes, fatty acids are imported into mitochondria via acylcarnitine intermediates, converted to acyl-CoA thioesters, then oxidized via β-oxidation or re-esterified into triglycerides and stored [15] (Fig 1). Importantly, a prolonged bioenergetic shift away from fatty acid oxidation is a well-described characteristic in the etiology of heart failure [16], suggesting that this shift might contribute to the cardiomyopathy seen in FA [7]. A conditional gene-targeting approach has led to the development of frataxin-deficient animal models, which reproduce some pathophysiological features of FA, such as cardiac hypertrophy without skeletal muscle involvement, and large sensory neuron dysfunction without alteration of the small sensory and motor neurons [17]. Frataxin-deficient cells from these animal models exhibited mitochondrial dysfunction together with an abnormal abundance of lipid droplets, which suggested a defect in one (or more) of the many steps of lipid homeostasis and metabolism in FA (Fig 1) [17]. In addition, Chen *et al*. discovered that the iron/sphingolipid/3-phosphoinositide dependent protein kinase-1 (PDK1)/myocyte enhancer factor-2 pathway (Mef2) was activated in Drosophila melanogaster upon loss of frataxin [18]. In a follow-up study, they showed that loss of frataxin in the nervous system in mice also activates an iron/sphingolipid/PDK1/Mef2 pathway. Furthermore, sphingolipids and PDK1 activity were up-regulated in heart tissues from FA patients [19].

Decreased high-density lipoprotein (HDL) and apolipoprotein A-I (ApoA-l) levels in the general population are associated with an increased risk of mortality from ischemic cardiomyopathy and heart failure [20,21]. Cardiomyopathy and heart failure are also risk factors that are elevated in FA [6–8,22]. Unfortunately, there are conflicting data on HDL and ApoA-I levels in FA. Two studies have shown a decrease in HDL-cholesterol [6,23] compared with controls; whereas, two studies showed there was no difference between FA and controls [24,25]. One of these latter studies also showed that there was no difference in serum ApoA-I levels in FA when compared with controls [25]. However, the immunoassay used in this study could have suffered from typical problems encountered with immunoassay procedures such cross-reactivity with unknown serum proteins and/or competition with ApoA-I autoantibodies in the serum, which would lead to an underestimate of the ApoA-1 concentrations. In view of the known presence of serumApoA-I antibodies in cardiovascular disease [26,27], this seems to be the most likely explanation. Therefore, when analyzing serum ApoA-I, it is critical to employ an identical internal standard in order to compensate for any losses that might occur during the analytical procedure [28].

The widely-used statin drugs, which increase levels of serum ApoA-I through inhibition of 3-hydroxy-3-methylglutaryl-coenzyme A (HMG-CoA) reductase [29], have had a significant therapeutic benefit by reducing the risk for cardiomyopathy [30] and heart failure [31]. Furthermore, a meta-analysis of 32,258 dyslipidemic patients included in 37 randomized studies revealed that the percentage increase in ApoA-I levels was virtually identical to the increase of HDL levels at all doses of three statins (rosuvasatin, atorvastatin and simvastatin) that had been administered [29]. This raised the potential for a possible intervention in FA if serum ApoA-I levels are in fact reduced in FA patients when compared with normal subjects. Therefore, we have employed a highly specific assay based on stable isotope dilution ultraperformance liquid chromatography-multiple reaction monitoring/mass spectrometry (UPLC-MRM/MS) [28] to compare serum ApoA-I concentrations in FA cases with control unaffected subjects.

## Materials and methods

### Reagents and chemicals

All reagents and solvents were LC-MS Optima grade (Fisher, Hampton, NH) quality unless otherwise noted. The complete EDTA-free protease inhibitor cocktail was from Roche. Human ApoA-I standard, isolated from human HDL was from ProSpec (East Brunswick, NJ). [^13^C_6_^15^N_2_]-lysine and [^13^C_9_^15^N_1_]-tyrosine used for stable isotope labeling by amino acids in cell culture (SILAC) were from Cambridge Isotope Laboratories (Tewksbury, MA). Mouse serum, Triton X-100, NP-40, DL-dithiothreitol (DTT), iodoacetamide (IAA), diisopropylethylamine (DIPEA), 1,1,1,3,3,3-hexafluoro-2-propanol (HFIP), 5-sulfosalacylic acid (SSA), mevalonic acid (MVA), mevalonic acid 5-phosphate (MVA-5P), mevalonic acid 5-pyrophosphate (MVA-5PP), isopentenyl pyrophosphate (IPP), geranyl pyrophosphate (GPP), farnesyl pyrophosphate (FPP), and geranylgeranyl pyrophosphate (GGPP) were purchased from Millipore Sigma (St. Louis, MO). 2-mecaptoethanol (BME) was from BioRad (Hercules, CA). Ammonium bicarbonate (ABC), Dulbecco's phosphate-buffered saline (DPBS) was purchased from Fisher Scientific (Hampton, NH). Colloidal Coomassie, NuPAGE lithium dodecyl sulfate (LDS) sample buffer (4X), and sequencing-grade modified trypsin were from Promega (Madison, WI). LC grade water and acetonitrile were from Burdick and Jackson (Muskegon, MI).

### Cell lines and cell culture conditions

Roswell Park Memorial Institute (RPMI) medium and fetal bovine serum (FBS) were obtained from Sigma. Human kidney HEK293 cell line (ATCC CRL-1573) and human liver HepG2 cell line (ATCC HB 8065) were obtained from the American Type culture collection (ATCC). Cultured cells with a passage number of 10–20 were used in the experiments to reduce variability due to long-term culture conditions. Cells were maintained at 37 °C in a humidified atmosphere containing 5% CO_2_. The expression of SILAC-labeled ApoA-I protein was performed in HEK 293 cell line [28]. Briefly, pRK5 plasmid containing ApoA-I sequence was constructed, confirmed by DNA sequencing, and expanded in *E*. *coli*. The ApoA-I pRK5 plasmid was transfected into HEK293 cells using FuGENE 6 transfection reagent (Promega) following manufacture’s instruction. HEK293 cells were cultured in DMEM/F12/SILAC medium (Athena Enzyme systems) containing [^13^C_6_^15^N_2_]-lysine and [^13^C_9_^15^N_1_]-tyrosine. Transfected cells were harvested in NP-40 lysis buffer (137.5 mM NaCl, 50 mM Tris/HCl pH 7.5, 1mM EDTA, 0.5% Triton x-100, 0.5% NP-40, 1mM DTT) containing protease inhibitor cocktail (Roche) after 24 h transfection. The lysate was centrifuged at 16,000 × g for 30 min at 4 °C. The supernatant was aliquoted and stored at -80 °C until analyzed. Frataxin knockdown and simvastatin treatment were conducted in HepG2 cell line. Monolayer cell cultures were maintained in RPMI medium, supplemented with 10% FBS, 1% Pen/strep and 1mM glutamine.

### Human subjects

Serum samples were from 190 subjects (95 controls, 95 FA cases) enrolled in an ongoing study of FA at the Children’s Hospital of Philadelphia. The demographic information is summarized in Table 1 and complete patient information is provided in S1 Table. All serum protocol samples were immediately aliquoted and frozen at -80 °C until analysis. The study was approved by the Institutional Review Board (Protocol # 800924) of the University of Pennsylvania and of the Children’s Hospital of Philadelphia.

**Table 1:** Demographics for study cohort of 95 cases and 95 controls.

	Total	Gender		GAA1 repeat				Age of onset			
		M	F	GAA1 (<366)	GAA1 (367–545)	GAA1 (546–742)	GAA1 (>742)	3–8	9–12	13–18	>19
Controls	95	50	45								
Cases	95	45	50	23	24	21	27	25	21	30	19

### Sample preparation for ApoA-I quantification

Recombinant SILAC ApoA-I internal standard was spiked into all samples to adjust for any bioanalytical variability. All samples (serum and cell medium) were thawed at room temperature. A 10 μL of serum sample was diluted 5 times with DPBS. To each 5 μL of this dilution, 5 μL of SILAC-ApoA-I, 3 μL of BME and 7 μL of NuPage LDS sample loading buffer (5x) were added. All samples were loaded on NuPage Novex 10% Bis-Tris gels (Promega, Madison, WI) for protein separation. The samples were cut from gels, followed by reduction, alkylation, and tryptic digestion. The digested samples were extracted by addition of 50% acetonitrile with 3% formic acid and bath sonication for 20 min. An extra step of desalting and concentration was carried out on Macrospin column (The Nest Group Inc, Southborough, MA) according to manufacturer’s instruction. The eluted peptides were dried and re-suspended with 50 μL of mobile phase A.

### Quantification of ApoA-I by UPLC-MRM/MS analysis

Eight tryptic peptides that the spanned sequence of ApoA-I from D^13^ to K^238^ were chosen for analysis (D^13^LATVYVDVLK^23^, D^28^YVSQFEGSALGK^40^, L^46^LDNWDSVTSTFSK^58^, V^97^QPYLDDFQK^106^, W^108^QEEMELYR^116^, T^161^HLAPYSDELR^171^, L^189^AEYHAK^195^, and V^227^SFLSALEEYTK^238^) [28]. Quantification was conducted by determining the ratio of the sum of the peak areas from the three most intense MRM/MS signals with the sum of three most intense signals from the corresponding [^13^C^15^N]-ApoA-I-derived tryptic [^13^C^15^N]-peptide. [^13^C^15^N]-ApoA-I, prepared using stable isotope labeling by amino acids in cell culture (SILAC) [28] was used as the internal standard. It was added to each calibration standard, quality control (QC) sample (22.5, 200.0 and 400.0 mg/dL), and serum sample before loading samples on the sodium dodecyl sulfate-polyacrylamide gel electrophoresis (SDS-PAGE) gel. In-batch QCs were analyzed on the SDS-PAGE gel along with the FA case samples, and run in the queue every 50 samples. Serum concentrations were determined by interpolation of the area ratios with a standard curve prepared in mouse serum at the same time by adding known amounts of ApoA-I to a fixed amount of the ApoA-I SILAC-standard. UPLC-MRM/MS was performed on a TSQ Vantage (Thermo Scientific, San Jose CA) equipped with a CaptiveSpray^™^ ion source (Michrom Bioresources, Auburn, CA). The mass spectrometer was interfaced with a nanoAcquity UPLC system (Waters Corporation, Milford, MA) equipped with an autosampler and sample thermo-controller (set at 4 °C). Both the UPLC and mass spectrometer were controlled by Xcalibur software (Thermo Scientific). Separations were performed using a Waters BEH130 C18 column (150 μm x 100 mm, 1.7 μm, 130 Å) at 30 °C using a partial loop injection. Samples were eluted with a linear gradient at a flow rate of 2 μL/min. Solvent A was water/acetonitrile (99.5:0.5, v/v) containing 0.1% formic acid, and solvent B was acetonitrile/water (98:2, v/v) containing 0.1% formic acid. The UPLC gradient was as follows: 95% A for 5 min, linearly decreased to 40% A over 35 min, 5% A at 36 min, held to 45 min, then re-equilibrate at 95% A from 46 to 55 min. The MS operating conditions were as follows: spray voltage, 1800 V; ion transfer capillary temperature, 270 °C; collision gas, argon at 1.5 mTorr; ion polarity, positive; scan type, multiple reaction monitoring; chrom filter peak width, 15 s; S-lens, 127 v; cycle time, 1.5 sec; Q1 peak width (FWHM), 0.7 u; Q3 peak width, 0.7 u; dissociation voltage (DCV), 10 V.

### Frataxin knockdown and simvastatin treatment in HepG2 cells

Small interfering (si)RNAs (sFXN#9, sFXN#10, sFXN#11 and sFXN#12) targeting human FXN and the corresponding negative control siRNA (si-NC) were obtained from QIAGEN. Target sequences of the four siRNAs were as follows, AGGAACCTATGTGATCAACAA (sFXN#9), CAACCAGATTTGGAATGTCAA (sFXN#10), AACGCTGGACTCTTTAGCAGA (sFXN#11), and TCCTTTGGGAGTGGTGTCTTA (sFXN#12). All siRNAs were prepared in RNAase free water as a working concentration at 10 μM. Knockdown effects from 16–24 h by the four siRNAs were analyzed in preliminary experiments by western blot. The sFXN#9 and sFXN#12 cells had obvious growth defects and so were not investigated further.

A stock solution of simvastatin was dissolved in dimethyl sulfoxide (DMSO) and then diluted using culture media such that the final concentration of DMSO added to cells did not exceed 0.1%. Control cells were treated with 0.1% DMSO. The doses of simvastatin used in this study relate closely to blood levels of simvastatin in patients treated with 10–80 mg of the drug. Biological samples were prepared in triplicates. Reverse transfection involving simultaneously transfecting and plating cells was performed using Lipofectamine RNAiMAX according to the manufacture protocol (Invitrogen). Briefly, the transfection mix including 30 pmol siRNA and transfection reagent was made and added to the 6-well plates. After 16 h knockdown by siRNAs, medium was changed to fresh and cells were cultured another 24 h to get 70% frataxin knockdown effect. The knockdown cells were re-plated in a new 6-well plate, and treated by different doses of simvastatin. After 48 h simvastatin treatment, medium was taken out for analysis of ApoA-I. Cell pellets were used for analysis of intermediates of MVA pathway. Total protein content was estimated using Bradford assay.

### Western blot analysis

Equal amounts of proteins were resolved on SDS-PAGE gel and then transferred to nitrocellulose membranes using a Mini Trans-Blot cell (Bio-Rad). After blocking with 5% non-fat milk, the membranes were incubated overnight with the following primary antibodies, mouse anti-frataxin (Abcam, Cambridge, UK, ab113691). Afterwards, the membranes were developed with horse radish peroxidase (HRP)-conjugated secondary antibodies using a chemiluminescent substrate (Bio-Rad). Densitometry was performed for semi-quantification of signals on membranes on an ImageQuant LAS4000 densitometer (GE healthcare Life Sciences).

### Sample preparation for HMG-CoA and MVA intermediates from HepG2 cells

After frataxin knockdown and treatment with simvastatin, HepG2 cells were washed with ice cold 0.9% NaCl buffer and harvested in 1 mL ice-cold methanol/water (4:1) solvent followed by pulse-sonication for 30 seconds on ice using a sonic dismembranator (Fisher), followed by a 10-min centrifugation at 15,000 x g. The supernatant was transferred to a clean tube, and evaporated to dryness under nitrogen and resuspended in 50 μL of 5% SSA in methanol/water (1:1) for analysis of HMG-CoA and intermediates of MVA pathway.

### Quantification of HMG-CoA and MVA intermediates by UPLC-high resolution (HR)MS

Acyl-CoAs including HMG-CoA and MVA intermediates were analyzed on an Ultimate 3000 Quaternary UPLC coupled with a Q Exactive Plus mass spectrometer operating in the negative ion mode with a heated electrospray ionization (ESI) probe in an Ion Max source housing. Samples were kept in a temperature-controlled autosampler at 4 °C. UPLC separation was performed on a Waters XSelect HSS C18 2.1 × 150 mm column at a flow at 0.2 mL/min. UPLC conditions modified from our previous study [32] were as follows: column oven temperature 55 °C, solvent A was water with 5 mM DIPEA and 200 mM HFIP, solvent B was methanol with 5 mM DIPEA and 200 mM HFIP. The UPLC gradient was as follows: 100% A for 6 min, 98% A at 8 min, 60% A at 10 min, 20% A at 16 min, held to 25 min, then re-equilibrated at 100% A from 26 to 35 min. Operating conditions on the mass spectrometer were as follows: spray voltage 4.5 kV, capillary temperature 320 °C, sheath gas 20 arbitrary units (arb), auxiliary gas 5 arbitrary units, S-lens RF-level 50, auxiliary gas heater temperature 250 °C, and in-source CID 5 eV. Two full scans ranged from 70 to 600 m/z and 750–1000 m/z were set to corresponding retention time for intermediates of MVA pathway and Acyl-CoAs metabolites, respectively, at 60,000 resolution and at 1e6 automatic gain control (AGC). Selective ion scan (full MS/SIM) was set with an inclusion list of all precursors at a resolution of 120, 000 (at m/z 200) and at 8e5 AGC.

### Data and statistical analysis

Analysis of ApoA-I chromatograms was performed in Skyline (MacCoss Laboratory). The three most intense MRM transitions of each peptide were selected based on the UPLC-tandem MS spectra on a TSQ Vantage. The peptide ratios were calculated by the total light (unlabeled)/heavy (labeled) peptide ratios of three MRM/MS transitions. The ApoA-I protein ratio was calculated by the average of eight selected peptides. Serum concentrations of ApoA-I were obtained by interpolation from a standard curve prepared in surrogate matrix. Data analysis of HMG-CoA and intermediates of MVA pathway were processed in Xcalibur and TraceFinder (Thermo Scientific). Statistical analysis was performed using GraphPad Prism (v 5.01, GraphPad Software Inc., La Jolla, CA) and Stata (v 15, Stata Corp, College Station, TX). Descriptive statistics were computed for all study variables including mean, standard deviation (SD), and range for continuous variables and proportion for categorical variable. Nonparametric Wilcoxon (Mann-Whitney) rank sum test was used for comparison using p-value of 0.05 as cutoff for a statistical significant result. Differences in mean ApoA-I between FA cases and controls were evaluated using multiple linear regression models to adjust for age, gender, and GAA1 length in a separate linear regression analysis, trend in ApoA-I across GAA1 length (in roughly four equal-size groups) in FA cases was also tested. To determine the value of using ApoA-I to classify FA cases from controls, an ROC analysis was performed to estimate the area under the ROC curve (AUC) and the associated confidence interval. A cutoff value of ApoA-I that maximized the accuracy (i.e., sum of sensitivity and specificity-1) was determined. Comparisons of ApoA-I level and HMG-CoA in 0.1% DMSO or simvastatin treated groups were made using two-way ANOVA models.

## Results

### Serum ApoA-I levels were reduced in FA

**Fig 2:**
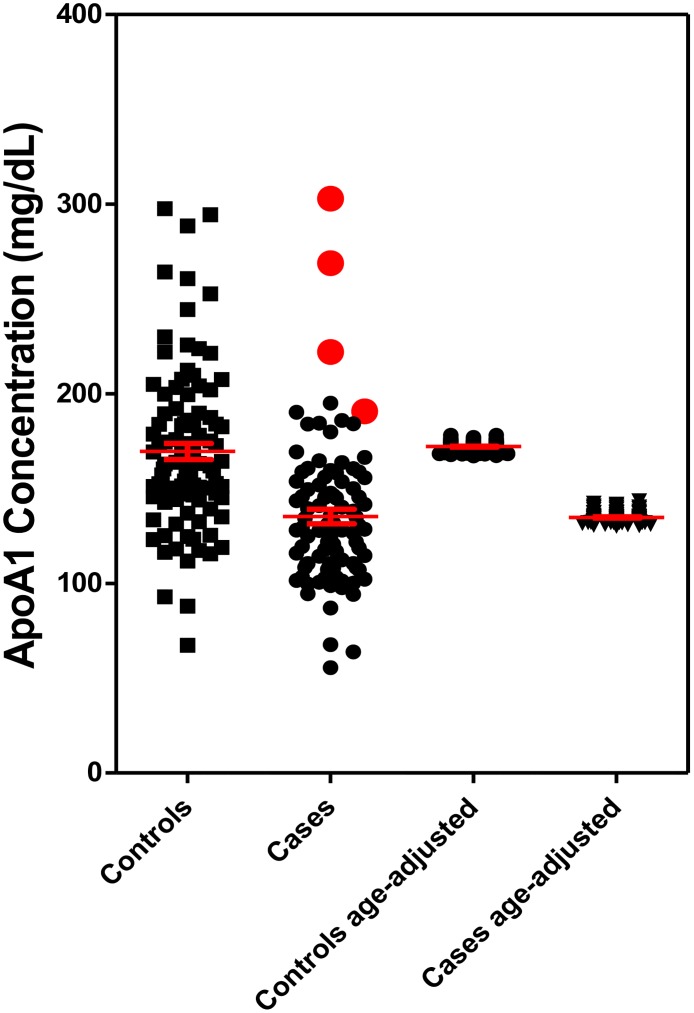
Serum ApoA-I concentrations in 95 controls and 95 cases determined by stable isotope dilution by UPLC-MRM/MS. Controls, mean = 172.1 ± 40.2 mg/dL; Cases mean 134.8 ± 37.1 mg/dL. The samples shown in red were re-analyzed and identical values were obtained.

A total of 190 serum samples (95 controls and 95 FA patients) were processed using a validated UPLC-MRM/MS method. All ApoA-I concentrations were within the linear range of standard curves. The mean concentrations (± SD) of ApoA-I in controls and FA patients were 172.1 mg/dL (± 40.2) and 134.8 mg/dL (± 37.1), respectively (Fig 2).

**Fig 3:**
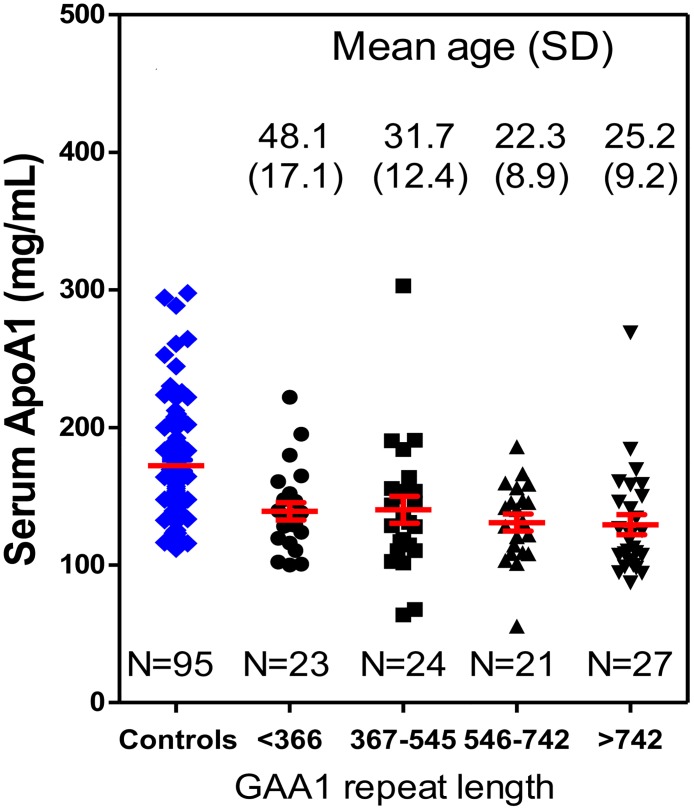
Analysis of serum ApoA-I concentrations. Relationship with GAA1 repeat length—95 controls, 23 FRDA cases 1st quartile, 24 FRDA cases 2nd quartile, 21 FRDA cases 3rd quartile, 27 FRDA cases 4th quartile.

**Fig 4:**
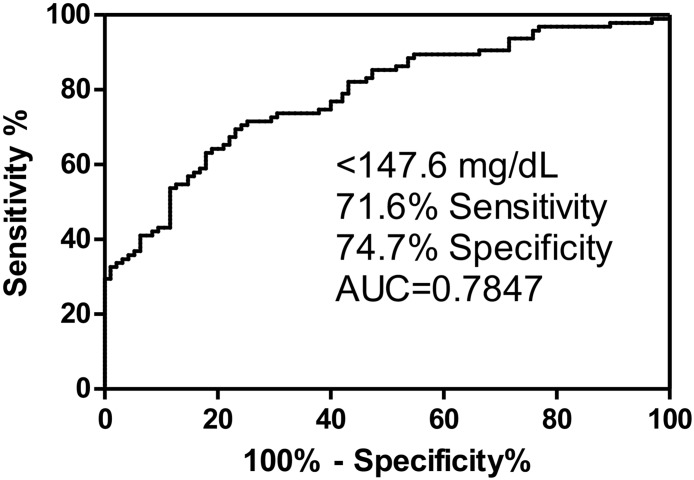
Receiver operator characteristic (ROC) curve for ApoA-I. The curve was generated from ApoA-I levels in FA cases (n = 95) versus ApoA1 levels in controls (n = 95).

All eight tryptic peptides showed the same trends in both groups. The FA patients on average had statistically significant lower serum ApoA-I concentrations compared with controls (21.6% reduced, rank sum test, p< 0.001). The mean reduction in ApoA-I concentration between cases and controls remained statistically significant at 36.4 mg/dL (p<0.001) after adjusting for age and sex. Among all the samples, four cases showed abnormally high level of serum ApoA-I, which fell into the high range of control ApoA-I concentration. Re-analysis of the serum samples from these FA patients one-year after the initial analysis revealed almost identical values (S2 Table) showing that samples do not deteriorate on storage and showed that the assay is very robust. Although ApoA-I levels increased with age, serum ApoA-I levels among cases were not correlated with GAA1 length (Fig 3). Unadjusted correlation between GAA1 length and ApoA-I levels was -0.12 (p = 0.26) and the age-adjusted partial correlation was -0.11 (p = 0.31) and the sex adjustment did not make any difference, -0.11 (p = 0.32). After adjusting for age, sex, and GAA1 length, mean ApoA-I concentrations for cases remained to be lower than controls (mean difference: 30.5 mg/dL, 95%CI: 8.6 mg/dL to 52.2 mg/dL, p = 0.006). The generation of a receiver operating characteristic (ROC) curve by ApoA-I in FA patients (N = 95) and ApoA-I in controls (N = 95) revealed an area under the curve (AUC) of 0.78 (95% CI: 0.72–0.85). When the cut-off set as 147.6 mg/dL, the ROC curve gave 71.6% sensitivity and 74.7% specificity (Fig 4).

### ApoA-I secretion was reduced with frataxin knockdown in HepG2 cells

**Fig 5:**
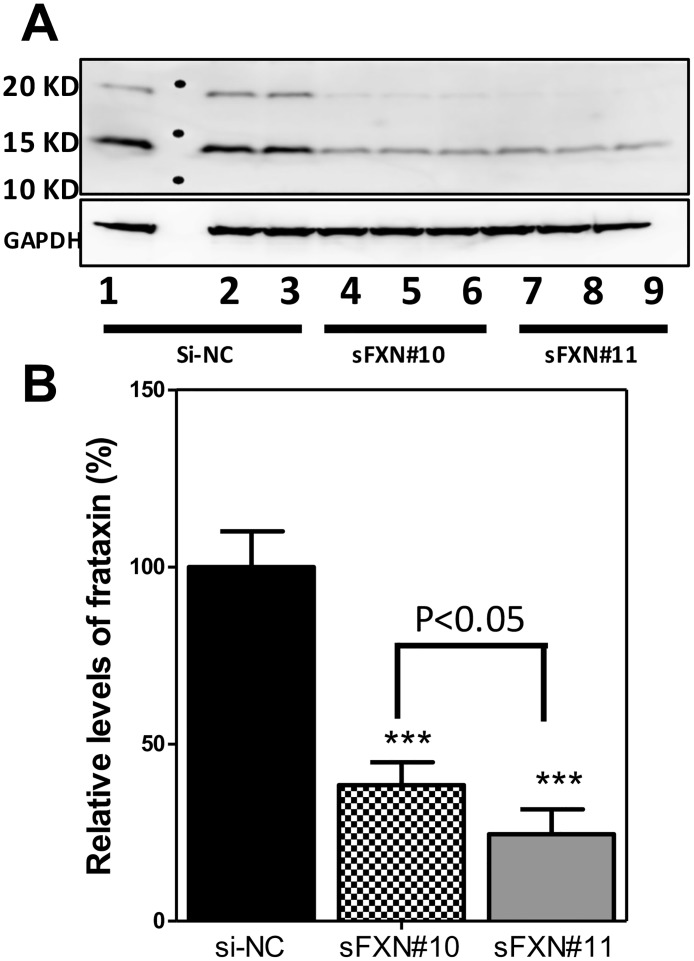
Analysis of frataxin knockdown in HepG2 cells. (A) Western blot analysis for verification of frataxin knockdown. (B) Quantitative analysis of western blots by Image J. Samples 1–3, treated with negative control siRNA (si-NC); Samples 4–6, treated with sFXN#10; Samples 7–9, treated with sFXN#11. GAPDH was used as a loading control. ***P < 0.001.

**Fig 6:**
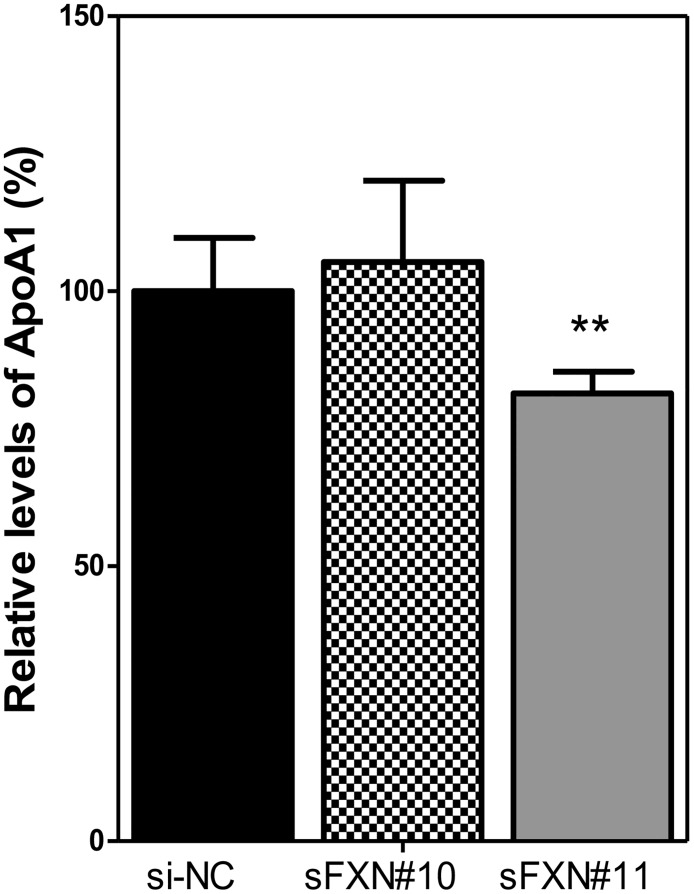
ApoA1 secreted by frataxin knockdown HepG2 cells. Levels (means ± SD; n = 3) were normalized to cellular protein concentration. **P < 0.01.

We did not observe a time-dependent depletion of protein frataxin pools from whole cell lysate for all tested siRNAs, thus for convenience we used the 16 h time-point. Of the siRNAs tested, sFXN#11 resulted in the strongest frataxin knockdown among five siRNAs. Image quantification was conducted only on the biologically active 14.3 kDa mature frataxin (81–210). After 16 h knockdown by sFXN#11, mature frataxin expression was reduced by 76% when compared with control cells (P < 0.001, Fig 5B). In sFXN#10 cells, frataxin expression was reduced by 62% compared with the control cells (P < 0.001, Fig 5B). However, the knockdown was significantly less than for sFXN#11 (P < 0.05, Fig 5B). There was a significant 23% decrease in ApoA-I secretion when compared with the control cells (p < 0.01, Fig 6). In contrast, for sFXN#10 where frataxin was reduced by 62% compared with the control group (Fig 5B), there was no effect on ApoA-I protein secretion (Fig 6).

### ApoA-I was increased by simvastatin treatment of frataxin knockdown HepG2 cells

**Fig 7:**
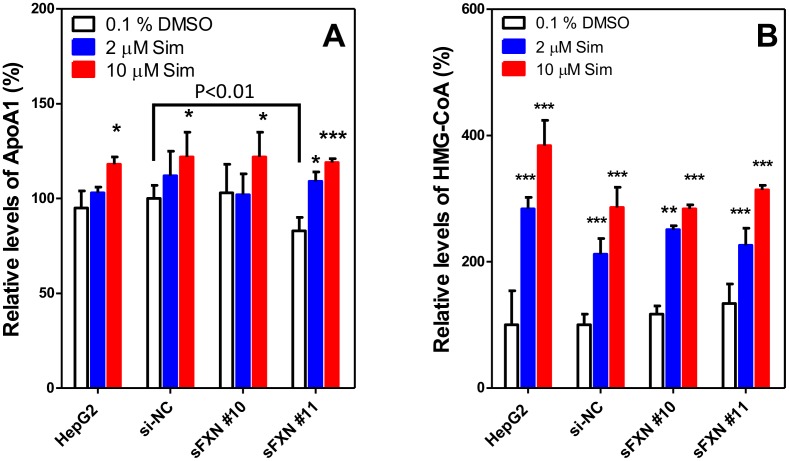
ApoA-I and HMG-CoA levels in HepG2 cells after treatment with simvastatin. (**A)** Relative levels of secreted ApoA1. (**B)** Relative HMG-CoA levels. Levels (means ± SD; n = 3) were normalized to cellular protein. P values are differences between simvastatin treated group and 0.1% DMSO treated group for each siRNA. *P < 0.05, **P < 0.01, ***P < 0.001. Cells were treated with simvastatin (SIM) was for 48 h.

There was a significant decrease in ApoA-1 expression between the sFXN#11 knockdown cells and the control si-NC cells (P<0.01, Fig 7A). In the sFXN#11 knockdown cells, an increase of secreted ApoA-I was observed with treatment of 2 μM simvastatin (109.4 ± 5.1%, P < 0.05, Fig 7A), and a greater effect was obtained with treatment of 10 μM simvastatin (119.2 ± 2.3%, P < 0.001, Fig 7A) when compared to the sFXN#11 DMSO group (83.3 ± 7.2%, Fig 7A). In the si-NC knockdown group, regular HepG2 group and sFXN#10 group, a significant increase of ApoA-I was only detected in 10 μM simvastatin treated cells (122.3 ± 12.9%, P < 0.05 in si-NC group; 118.2 ± 9.4%, 122.2 ± 13.0% P < 0.05 in regular HepG2 group, and P < 0.05 in sFXN#10 group). A higher dose of simvastatin was tested but it did not have a further stimulatory effect on ApoA-I levels.

### Modulation of HMG-CoA and MVA metabolite formation by frataxin knockdown

Baseline levels of HMG-CoA (determined by UPLC-HRMS) in sFXN#10 group (117.4 ± 17.2%, Fig 7B) and sFAN#11 knockdown group (134.3 ± 31.1%, Fig 7B) showed a trend to higher levels than the si-NC group (100.0 ± 17.3%, Fig 7B) and in regular HepG2 group (100.4 ± 54.3%, Fig 7B). However, the differences did not reach statistical significance. HMG-CoA demonstrated a dramatic increase after treatment with 2 μM simvastatin in all groups (average 243.3 ± 31.6%). A greater increase of HMG-CoA was observed after treatment with 10 μM simvastatin (mean 317.0 ± 46.7%). We also quantified MVA and its downstream metabolites by UPLC-HRMS. Basal levels of MVA, MVA-5P, MVA-5PP and IPP were detectable before treatment with simvastatin at very low levels (< 3 pmol/mg protein) as described previously [33]. However, after simvastatin mediated inhibition of HMG-CoA, they were below the limit of detection of the UPLC-MS assay (<0.2 nmol/mg protein) in the control HepG2 cells as well as the frataxin knockdown cells.

## Discussion

There are currently no approved treatments for FA although numerous therapeutic approaches are currently under development [34]. While there there is no necessity to develop biomarkers for disease diagnosis because it is readily identified from GAA-repeat analysis, there are no reliable biomarkers ailable to monitor the effect of new therapies. We have recently implemented a method that examines the ability of platelets to metabolize labeled glucose and palmitate to acyl-CoA metabolites. Unfortunately, this method is very labor-intensive and requires sophisticated sample collection techniques that are not available in many centers treating FA. Therefore, we have developed an alternative approach that could have wider utility. Our platelet data indicated that there could be dysregulation of HMG-CoA biosynthesis, which could result in downstream down-regulation of ApoA-I through the PPARα pathway (Fig 1). This observation, together with the finding that decreased ApoA-l levels in the general population are associated with an increased risk of mortality from cardiomyopathy and heart failure [20,21], led us to compare serum Apo-AI levels in FA with a large cohort of control subjects.

The levels of serum ApoA-I found in the control subjects (172.1 mg/dL, Fig 2) was almost identical to the value we obtained for a completely separate cohort of controls (170.4 mg/dL) [28]. It is difficult to compare these values to those obtained previously by ELISA methodology in control subjects because such assays are unable to detect differences in serum ApoA-I between non-smokers and tobacco smokers [35], another population at risk for heart failure [36]. Furthermore, in a large study, the widely used statin Lipitor (atorvastatin) was found by ELISA to cause a decrease rather than the excpeted increase in serum ApoA-I levels with increasing doses [29]. Smoking cessation is known to reduce the risk for heart failure [37] and a meta-analysis of 27 studies revealed that HDL levels were elevated upon smoking cessation [38]. As ApoA-I is a major component of HDL, our more specific stable isotope dilution UPLC-MRM/MS assay should reveal an increase in serum ApoA-I in smokers who quit. In addition, we anticipate that the assay will provide much more reliable data on serum ApoA-I levels in FA cases in the future. The 21.6% reduction in serum ApoA-I that we found in FA is very similar to the reduction levels (20.2%) that we found in tobacco smokers. There was no correlation with GAA repeat length (Fig 3) but the ROC curve discriminated cases from controls with an AUC of 0.78 (Fig 4).

Unfortunately, ApoA-I biosynthesis is not regulated by PPARα in rodents [39], which meant that a human cell line had to be used to test the *in vitro* effects of statins rather than the numerous model rodent FA cell lines that are available. Furthermore, ApoA-I is only synthesized in liver and intestinal cells [40] and so human hepatoma HepG2 cells have provided a useful model system to test the effect of statins on ApoA-I biosynthesis [41–43]. Consequently, HepG2 cells were employed in the present study to test whether frataxin knockdown combined with statin treatment could also have effects on ApoA-I biosynthesis. Statins are inhibitors of the early rate-limiting enzyme in cholesterol synthesis, HMG-CoA reductase, which catalyzes the conversion of HMG-CoA to MVA (Fig 1). Previous studies have revealed that atorvastatin, simvastatin and pitavastatin all increase the biosynthesis of ApoA-I in HepG2 cells [41–43]. Additional studies revealed that effects of statins on apoA-I biosynthesis were abolished by MVA, the immediate downstream metabolite of HMG-CoA (Fig 1) [41]. In contrast, inhibition of geranylgeranyl transferase activity or treatment with an inhibitor of the Rho GTP-binding protein family increased ApoA-I biosynthesis through activation of PPARα [41].

Frataxin protein has not previously been analyzed in HepG2 cells. We found that it was primarily the mature 14.3 kDa mitochondrial form of frataxin (81–210) by specific SDS-PAGE western blot (Fig 5A) and proteomic analysis. This form of frataxin was expressed at a level similar to that found in human HEK293 kidney cells, SH-SY5Y neuronal cells, HeLa cells, and dermal fibroblasts [44,45]. We also found that HepG2 cell frataxin was readily down-regulated by siRNA techniques (Fig 5B) to give levels of frataxin found in FA patients (Fig 2), which are typically > 70% lower than in normal cells [46]. Changes in ApoA-I secretion with or without treatment with simvastatin in HepG2 cells were determined by UPLC-MRM/MS. Cells with a frataxin knockdown of > 70% secreted ApoA-I at a level that was 23% lower than the control HepG2 cells. Interestingly, when frataxin was only reduced to levels that were 62% of normal values in the HepG2 cells, there was no effect on ApoA-I secretion (Fig 6).

Statin treatment caused a dose-dependent increase in ApoA-I secretion in both frataxin knockdown and control HepG2 cells (Fig 7A). There was a concomitant increase in HMG-CoA (Fig 7B) together with a decrease in the immediate downstream metabolites of MVA to levels that could not be detected by UPLC-MS. These data are in keeping with a statin-mediated reduction of geranylgeranylation of RhoA, which results in increased expression of PPARα (a transcription factor for ApoA-I) followed by increased transcription of ApoA-I (Fig 1) as described by Martin *et al*. [41].

Although statins can only up-regulate ApoA-I expression in hepatic and intestinal tissues, the concomitant increase in HMG-CoA levels would occur in all tissues. The potential therapeutic benefit in neurodegenerative disease has not been explored in detail. However, simvastatin reversed the cardiac hypertrophy and fibrosis and improved cardiac function in a transgenic rabbit model of human hypertrophic cardiomyopathy [47].

### Conclusion

The finding that ApoA-I is reduced in FA (a disease with reduced frataxin expression) and that statins can induce the biosynthesis of ApoA-I in a cellular model of frataxin deficiency suggests that statins could be used to increase serum ApoA-I levels in FA patients. Our *in vitro* model system revealed that there might be a threshold frataxin level where statins would not be effective. Furthermore, the ApoA-I levels did not correlate with GAA repeat length. Therefore, it will be important in the future to assess frataxin levels in particular FA cases in order to determine whether there is a threshold level required for optimal ApoA-I secretion. We have recently developed an assay to rigorously quantify mature frataxin in human platelets as surrogates for liver tissue so that such determinations can be readily made [48]. Interestingly, there were four FA patients with serum ApoA-I levels that were in the normal range and repeat analyses confirmed that this was not due to assay specificity. It will be interesting to determine whether the elevated levels in these patients persist and whether have a decreased risk for cardiovascular disease.

## Supporting information

S1 Table Individual demographics of 95 cases and 95 controls who provided serum samples. (DOCX)

S2 Table Re-analysis of the serum samples for FA cases with abnormal ApoA-I levels. (DOCX)
