# Supplementary material for: Low apolipoprotein A-I levels in Friedreich’s ataxia and in frataxin-deficient cells: Implications for therapy
Source: PLoS One. 2018 Feb 15;13(2):e0192779. doi: 10.1371/journal.pone.0192779 (PMC5813973; doi:10.1371/journal.pone.0192779)
Supplement: S1 Table — (DOCX) [file pone.0192779.s001.docx]

Low apolipoprotein A-I levels in Friedreich’s ataxia and in frataxin-deficient cells: Implications for therapy

**QingQing Wang^1,2^, Lili Guo^2^, Cassandra J. Strawser^1,3^, Lauren A. Hauser^1,3^, Wei-Ting Hwang^4^, Nathaniel W. Snyder^5^, David R. Lynch^1,3^, Clementina Mesaros^1,2^, Ian A. Blair^1,2,^***

^1^Penn/CHOP Center of Excellence in Friedreich’s Ataxia, The Children’s Hospital of Philadelphia, PA 19104, U.S.A., ^2^ Penn SRP Center and Center of Excellence in Environmental Toxicology Center, Department of Systems Pharmacology and Translational Therapeutics, Perelman School of Medicine, University of Pennsylvania Philadelphia, PA 19104, U.S.A., ^3^Division of Neurology, The Children’s Hospital of Philadelphia, PA 19104, U.S.A., ^4^Department of Biostatistics, Epidemiology, and Informatics, Perelman School of Medicine, University of Pennsylvania, Philadelphia, PA 19104, U.S.A., ^5^AJ Drexel Autism Institute, Drexel University, Philadelphia, PA 19104, U.S.A.

Corresponding author:

* [ianblair@upenn.edu](mailto:ianblair@upenn.edu)

**S1 Table: Full demographics data for study cohort of 95 comntrols and 95 cases**

|  |  |  |  |  |  |  |  |  |
| --- | --- | --- | --- | --- | --- | --- | --- | --- |
| **Controls** | | | **Cases** | | | | | |
| **Sex (M=1, F=2)** | **Age** | **ApoA1 (mg/dL)** | **Sex (M=1, F=2)** | **Age** | **Age of Onset** | **GAA1** | **GAA2** | **ApoA1 (mg/dL)** |
| 2 | 33 | 221.4 | 2 | 29 | 9 | 1000 | 1000 | 125.0 |
| 1 | 34 | 186.0 | 2 | 30 | 20 | 365 | 953 | 100.0 |
| 1 | 52 | 294.4 | 1 | 19 | 13 | 533 | 1500 | 110.5 |
| 1 | 22 | 244.4 | 2 | 54 | 17 | 533 | 1400 | 102.7 |
| 2 | 39 | 192.2 | 1 | 63 | 43 | 100 | 1000 | 123.8 |
| 1 | 41 | 202.0 | 2 | 9 | 5 | 546 | 546 | 153.8 |
| 1 | 19 | 204.9 | 2 | 12 | 7 | 546 | 546 | 114.7 |
| 1 | 35 | 133.4 | 1 | 46 | 35 | 136 | 1011 | 110.5 |
| 1 | 52 | 199.5 | 2 | 23 | 7 | 790 | 1120 | 107.3 |
| 2 | 26 | 152.6 | 1 | 24 | 5 | 800 | 800 | 117.9 |
| 1 | 52 | 174.5 | 1 | 19 | 15 | 366 | 733 | 119.4 |
| 2 | 22 | 264.2 | 2 | 26 | 8 | 750 | 750 | 87.1 |
| 2 | 64 | 151.9 | 2 | 60 | 43 | 41 | 696 | 164.7 |
| 1 | 53 | 170.2 | 1 | 39 | 16 | 466 | 833 | 117.0 |
| 2 | 43 | 117.4 | 2 | 44 | 23 | 450 | 670 | 155.7 |
| 1 | 16 | 230.1 | 1 | 22 | 12 | 521 | 775 | 110.5 |
| 1 | 37 | 123.2 | 2 | 33 | 17 | 533 | 900 | 153.7 |
| 1 | 70 | 172.6 | 1 | 31 | 15 | 454 | 777 | 135.7 |
| 1 | 49 | 157.1 | 2 | 19 | 12 | 700 | 700 | 101.2 |
| 1 | 62 | 116.4 | 1 | 30 | 22 | 733 | c.100delG | 131.7 |
| 1 | 26 | 172.6 | 2 | 17 | 10 | 486 | 953 | 118.6 |
| 1 | 62 | 164.4 | 2 | 30 | 18 | 252 | 972 | 152.0 |
| 1 | 21 | 151.4 | 2 | 10 | 5 | 760 | 1100 | 149.9 |
| 1 | 40 | 125.3 | 2 | 7 | 3 | 1000 | 1000 | 97.8 |
| 1 | 55 | 178.8 | 1 | 57 | 41 | 126 | 520 | 128.8 |
| 2 | 39 | 144.1 | 2 | 27 | 8 | 790 | 1120 | 94.6 |
| 2 | 32 | 161.9 | 2 | 46 | 43 | 166 | 833 | 222.0 |
| 2 | 22 | 123.3 | 2 | 17 | 15 | 400 | 800 | 101.6 |
| 1 | 19 | 161.5 | 2 | 33 | 12 | 809 | 809 | 126.5 |
| 2 | 20 | 189.7 | 1 | 20 | 12 | 841 | 841 | 94.2 |
| 1 | 47 | 135.1 | 1 | 20 | 16 | 333 | 733 | 130.5 |
| 2 | 28 | 147.6 | 1 | 24 | 13 | 525 | 1025 | 143.6 |
| 1 | 42 | 115.6 | 1 | 61 | 28 | 325 | 1025 | 129.2 |
| 2 | 54 | 183.9 | 1 | 71 | 41 | 90 | 1025 | 146.4 |
| 2 | 31 | 160.4 | 1 | 21 | 9 | 846 | 1000 | 145.8 |
| 1 | 26 | 149.3 | 1 | 22 | 17 | 550 | 750 | 109.0 |
| 2 | 51 | 142.8 | 1 | 28 | 18 | 300 | 550 | 129.0 |
| 2 | 35 | 111.8 | 1 | 45 | 5 | 662 | 860 | 145.5 |
| 2 | 31 | 169.5 | 1 | 34 | 16 | 244 | 1117 | 102.2 |
| 2 | 35 | 159.3 | 2 | 17 | 8 | 800 | 933 | 141.0 |
| 2 | 54 | 145.6 | 2 | 37 | 12 | 590 | 990 | 145.4 |
| 2 | 27 | 173.0 | 1 | 60 | 44 | 325 | 1025 | 116.0 |
| 2 | 18 | 147.1 | 2 | 20 | 16 | 500 | 1000 | 145.1 |
| 1 | 22 | 159.2 | 2 | 32 | 11 | 500 | 1250 | 131.1 |
| 1 | 50 | 150.8 | 1 | 16 | 8 | 833 | 4(p.G130V) | 110.2 |
| 2 | 39 | 187.5 | 1 | 12 | 11 | 699 | 996 | 141.7 |
| 1 | 61 | 137.1 | 2 | 34 | 17 | 533 | 900 | 190.7 |
| 2 | 47 | 164.0 | 1 | 35 | 8 | 690 | 1120 | 158.8 |
| 1 | 13 | 152.7 | 2 | 55 | 16 | 280 | 280 | 147.5 |
| 2 | 39 | 169.3 | 1 | 28 | 14 | 750 | 920 | 99.7 |
| 1 | 24 | 147.6 | 2 | 54 | 16 | 500 | 670 | 128.5 |
| 2 | 51 | 204.2 | 2 | 27 | 14 | 603 | 1001 | 108.4 |
| 2 | 40 | 175.8 | 2 | 40 | 19 | 376 | 992 | 127.9 |
| 1 | 49 | 145.2 | 2 | 49 | 16 | 480 | 740 | 163.8 |
| 2 | 57 | 139.3 | 1 | 21 | 10 | 650 | 950 | 128.1 |
| 1 | 56 | 154.0 | 1 | 27 | 7 | 750 | 850 | 107.2 |
| 2 | 57 | 189.4 | 2 | 28 | 8 | 721 | 943 | 120.5 |
| 2 | 54 | 260.8 | 1 | 78 | 63 | 114 | 1047 | 135.0 |
| 2 | 59 | 288.6 | 2 | 30 | 20 | 140 | 1005 | 138.1 |
| 2 | 58 | 222.0 | 2 | 59 | 9 | 350 | 750 | 160.6 |
| 1 | 51 | 225.7 | 2 | 21 | 12 | 637 | 1150 | 121.8 |
| 2 | 19 | 207.5 | 2 | 28 | 15 | 796 | 1129 | 184.2 |
| 2 | 29 | 209.7 | 1 | 17 | 8 | 742 | 1102 | 108.2 |
| 2 | 18 | 147.8 | 2 | 29 | 6 | 1000 | 1200 | 121.8 |
| 2 | 23 | 131.2 | 2 | 23 | 7 | 900 | 1000 | 169.4 |
| 1 | 37 | 144.3 | 2 | 45 | 18 | 500 | 1200 | 149.5 |
| 1 | 43 | 118.2 | 1 | 18 | 14 | 600 | 1000 | 146.8 |
| 2 | 21 | 155.7 | 2 | 17 | 11 | 633 | 800 | 159.5 |
| 2 | 14 | 163.2 | 1 | 44 | 11 | 800 | 800 | 112.4 |
| 1 | 27 | 159.4 | 1 | 23 | 10 | 787 | 1095 | 109.2 |
| 1 | 29 | 149.7 | 1 | 35 | 5 | 750 | 1000 | 134.2 |
| 1 | 49 | 124.7 | 2 | 11 | 8 | 833 | 900 | 98.8 |
| 1 | 49 | 125.4 | 2 | 19 | 15 | 533 | 733 | 190.3 |
| 2 | 27 | 203.4 | 1 | 27 | 16 | 659 | 1032 | 185.9 |
| 2 | 44 | 297.6 | 1 | 24 | 6 | 789 | 1305 | 158.7 |
| 2 | 31 | 150.3 | 1 | 36 | 18 | 233 | 1066 | 139.5 |
| 1 | 38 | 199.9 | 1 | 22 | 14 | 566 | 866 | 156.0 |
| 1 | 48 | 183.3 | 2 | 32 | 25 | 200 | 500 | 195.1 |
| 2 | 50 | 183.2 | 2 | 29 | 20 | 238 | 842 | 179.9 |
| 1 | 30 | 151.1 | 2 | 65 | 57 | 114 | 1033 | 128.1 |
| 1 | 42 | 150.2 | 1 | 68 | 47 | 237 | 900 | 100.7 |
| 2 | 22 | 151.0 | 1 | 32 | 10 | 925 | 1090 | 158.2 |
| 1 | 41 | 223.7 | 2 | 29 | 7 | 700 | 700 | 114.4 |
| 2 | 33 | 146.7 | 2 | 34 | 21 | 433 | 800 | 184.0 |
| 2 | 40 | 212.5 | 2 | 19 | 14 | 900 | 1300 | 268.9 |
| 1 | 22 | 173.9 | 2 | 47 | 7 | 760 | 860 | 160.7 |
| 1 | 24 | 132.5 | 2 | 15 | 8 | 570 | 790 | 166.4 |
| 2 | 36 | 162.9 | 2 | 22 | 13 | 486 | 836 | 303.0 |
| 1 | 50 | 182.6 | 1 | 18 | 5 | 769 | 1014 | 103.2 |
| 1 | 70 | 178.1 | 2 | 18 | 8 | 680 | 830 | 140.3 |
| 1 | 52 | 252.7 | 1 | 23 | 10 | 650 | 850 | 103.3 |
| 1 | 30 | 119.0 | 1 | 26 | 10 | 787 | 1095 | 108.4 |
| 2 | 28 | 207.6 | 1 | 17 | 12 | 466 | 666 | 67.7 |
| 1 | 45 | 183.9 | 1 | 43 | 15 | 500 | 570 | 63.8 |
| 1 | 52 | 175.0 | 1 | 18 | 10 | 700 | 1066 | 55.5 |
