# Supplementary material for: Low apolipoprotein A-I levels in Friedreich’s ataxia and in frataxin-deficient cells: Implications for therapy
Source: PLoS One. 2018 Feb 15;13(2):e0192779. doi: 10.1371/journal.pone.0192779 (PMC5813973; doi:10.1371/journal.pone.0192779)
Supplement: S2 Table — (DOCX) [file pone.0192779.s002.docx]

Low apolipoprotein A-I levels in Friedreich’s ataxia and in frataxin-deficient cells: Implications for therapy

**QingQing Wang^1,2^, Lili Guo^2^, Cassandra J. Strawser^1,3^, Lauren A. Hauser^1,3^, Wei-Ting Hwang^4^, Nathaniel W. Snyder^5^, David R. Lynch^1,3^, Clementina Mesaros^1,2^, Ian A. Blair^1,2,^***

^1^Penn/CHOP Center of Excellence in Friedreich’s Ataxia, The Children’s Hospital of Philadelphia, PA 19104, U.S.A., ^2^ Penn SRP Center and Center of Excellence in Environmental Toxicology Center, Department of Systems Pharmacology and Translational Therapeutics, Perelman School of Medicine, University of Pennsylvania Philadelphia, PA 19104, U.S.A., ^3^Division of Neurology, The Children’s Hospital of Philadelphia, PA 19104, U.S.A., ^4^Department of Biostatistics, Epidemiology, and Informatics, Perelman School of Medicine, University of Pennsylvania, Philadelphia, PA 19104, U.S.A., ^5^AJ Drexel Autism Institute, Drexel University, Philadelphia, PA 19104, U.S.A.

Corresponding author:

* [ianblair@upenn.edu](mailto:ianblair@upenn.edu)

**S2 Table: Re-analysis of the serum samples for FA cases with abnormal ApoA-I levels**

| Case | ApoA-I concentration (mg/dL) | | Mean (mg/dL) | Differ  (%) |
| --- | --- | --- | --- | --- |
|  | 1st measurement | 2nd measurement |  |  |
| 1 | 222.0 | 243.9 | 233.0 | -9.4% |
| 2 | 190.7 | 211.8 | 201.3 | -10.5% |
| 3 | 268.9 | 229.4 | 249.1 | 15.8% |
| 4 | 303.0 | 294.9 | 298.9 | 2.7% |
| Mean | 246.1 | 245.0 | 245.6 | 0.5% |
